# Influence of the Core/Shell Structure of Indium Phosphide Based Quantum Dots on Their Photostability and Cytotoxicity

**DOI:** 10.3389/fchem.2019.00466

**Published:** 2019-06-27

**Authors:** Karl David Wegner, Fanny Dussert, Delphine Truffier-Boutry, Anass Benayad, David Beal, Lucia Mattera, Wai Li Ling, Marie Carrière, Peter Reiss

**Affiliations:** ^1^Univ. Grenoble Alpes, CEA, CNRS, IRIG, SyMMES, STEP, Grenoble, France; ^2^Univ. Grenoble Alpes, CEA, CNRS, IRIG, SyMMES, CIBEST, Grenoble, France; ^3^Univ. Grenoble Alpes, CEA-LITEN L2N, Grenoble, France; ^4^Univ. Grenoble Alpes, CEA, CNRS, IRIG, IBS, Grenoble, France

**Keywords:** quantum dots, photostability, cytotoxcity, indium phosphide, core/shell structure, alumina coating

## Abstract

With the goal to improve their photostability, InP-based QDs are passivated with three types of inorganic shells, namely (i) a gradient ZnSe_x_S_1−x_ shell, (ii) an additional ZnS shell on top of the gradient shell with two different thicknesses (core/shell/shell, CSS), (iii) an alumina coating on top of ZnS. All three systems have photoluminescence quantum yields (PLQY) > 50% and similar PL decay times (64–67 ns). To assess their photostability they are incorporated into a transparent poly (methyl methacrylate) (PMMA) matrix and exposed to continuous irradiation with simulated sunlight in a climate chamber. The alumina coated core/shell system exhibits the highest stability in terms of PLQY retention as well as the lowest shift of the PL maximum and lowest increase of the PL linewidth, followed by the CSS QDs and finally the gradient shell system. By means of XPS studies we identify the degradation of the ZnS outer layer and concomitant oxidation of the emissive InZnP core as the main origins of degradation in the gradient structure. These modifications do not occur in the case of the alumina-capped sample, which exhibits excellent chemical stability. The gradient shell and CSS systems could be transferred to the aqueous phase using surface ligand exchange with penicillamine. Cytotoxicity studies on human primary keratinocytes revealed that exposure for 24 h to 6.25–100 nM of QDs did not affect cell viability. However, a trend toward reduced cell proliferation is observed for higher concentrations of gradient shell and CSS QDs with a thin ZnS shell, while CSS QDs with a thicker ZnS shell do not exhibit any impact.

## Introduction

**Figure 1:**
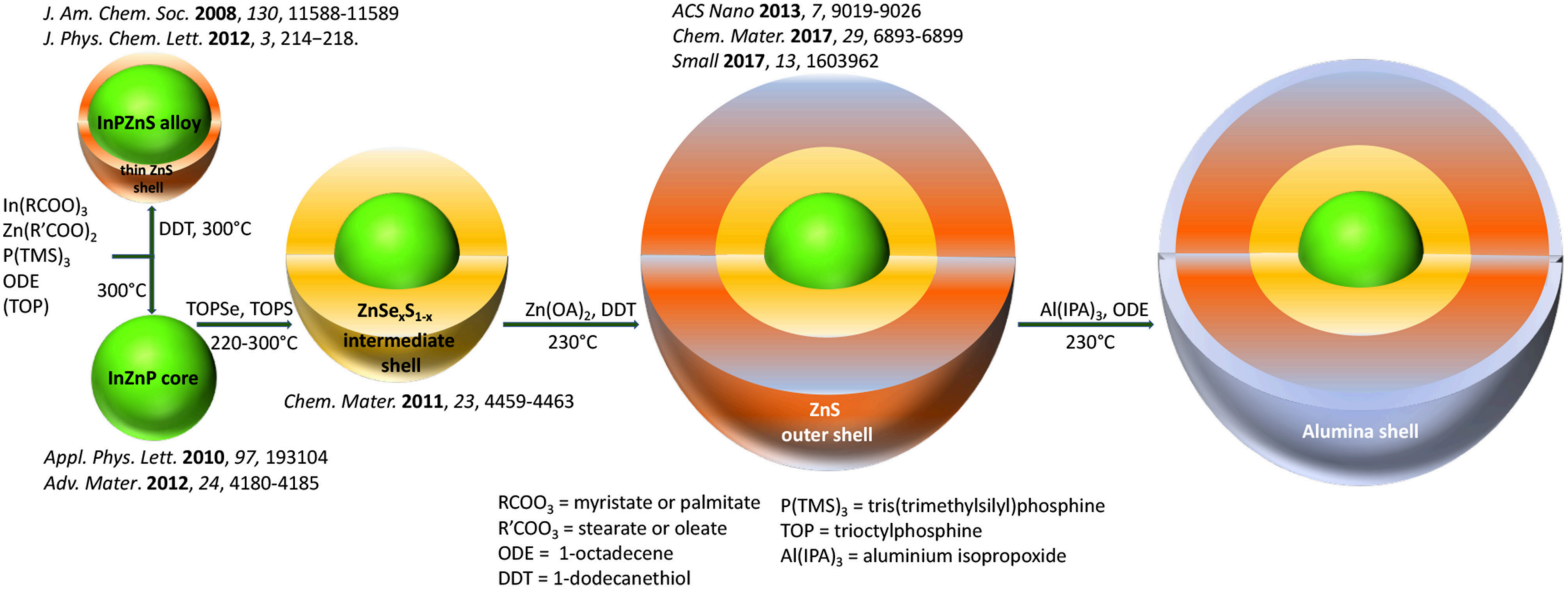
Strategies for the surface passivation of InP-based QDs, leading ultimately to the In(Zn)P/ZnSe_x_S_1−x_/ZnS core/shell/shell system, illustrated with representative references. The additional surface coating with an alumina shell as described in this work is also displayed in this scheme.

In the quest for toxic-heavy-metal-free quantum dots (QDs), indium phosphide has been shown to be a valid alternative to cadmium-based materials (Reiss et al., 2016; Allocca et al., 2019). The chemical synthesis of InP QDs and their core/shell structures has been strongly developed in the past decade with the goal to bring the optical properties to a level comparable with CdSe based QDs (Cossairt, 2016; Tamang et al., 2016). Concerning the core synthesis, it has been established that the addition of zinc, in form of carboxylate (e.g., stearate, undecylenate, oleate) improves both the size distribution and hence the emission linewidth and the emission efficiency (Li and Reiss, 2008; Xu et al., 2008; Ung et al., 2010b; Stein et al., 2016). Nonetheless, for achieving high photoluminescence quantum yields (PLQY) and enhanced photostability the precise engineering of core/shell heterostructures is a prerequisite, and ideally, the InP or InZnP core should be covered by a thick shell of a large band gap, chemically stable semiconductor such as ZnS (Reiss et al., 2009). The synthesis of InP/ZnS nanocrystals in a single-step one-pot method by adding a sulfur source (dodecanethiol, DDT) during the core synthesis leads to an alloy structure capped with a thin ZnS shell (Huang et al., 2010). Due to the large lattice mismatch of 7.7% between zinc blende InP and ZnS it is challenging to obtain a thick shell. ZnSe has a lower mismatch (3.3%) and several works reported the growth of either pure ZnSe or graded ZnSe_x_S_1−x_ shells on InP or InZnP (Lim et al., 2011; Tessier et al., 2015; Pietra et al., 2016; Chandrasekaran et al., 2017). However, ZnSe provides a weaker carrier confinement than ZnS due to the smaller band gap and is more sensitive to oxidation than ZnS (Toufanian et al., 2018). Therefore, it has been suggested to add a thicker outer ZnS shell on a graded intermediate ZnSe_x_S_1−x_ shell (Lim et al., 2013; Ramasamy et al., 2017; Wang et al., 2017). The latter acts as a lattice adapter between the core and the outer shell leading to a core/shell/shell (CSS) system, as already demonstrated in the case of II-VI semiconductors (Reiss et al., 2003; Talapin et al., 2004). Li et al. proposed another way to enhance the photostability of CdSe- or CuInS_2_- based QDs by generating an alumina coating on their surface (Li et al., 2015; Yan et al., 2016). In this case, the nanocrystal surface is treated with an appropriate aluminum precursor (e.g., aluminum isopropoxide), which transforms to alumina via oxidation. The different above-mentioned strategies for the surface passivation of InP and InZnP QDs are summarized in Figure 1.

The goal of the present work is to investigate the impact of the different core/shell systems discussed—core/gradient shell and CSS structures with different thickness of the outer ZnS shell and a newly developed alumina coating—on the photostability and cytotoxicity behavior of InZnP QDs. Aging experiments were carried out in a controlled and reproducible manner by integrating the QDs into a solid polymer matrix (PMMA) and irradiating them with simulated solar light in a climatic chamber. Finally, the cytotoxicity of the obtained QDs was assessed using human primary keratinocytes.

## Materials and Methods

### Chemicals

Indium acetate (99.99%), zinc acetate (99,9%), myristic acid (>99%), tris (trimethylsilyl)phosphine (95%, (TMS)_3_P), 1-octadecene (90%, ODE), trioctylphosphine (97%, TOP), sulfur (99.99%), selenium (99.99%), dimethylformamide (DMF), poly (methyl methacrylate) (PMMA, M_W_ ~350,000 GPC), aluminum isopropoxide (Al (IPA)_3_ 98%), toluene, methanol, chloroform, acetone, hexane, D-penicillamine, tetramethylammonium hydroxide (TMAOH), phosphate-buffered saline solution (1xPBS), and tris (2-carboxyethyl) phosphine hydrochloride solution 0.5 M (TCEP) were purchased from Sigma-Aldrich. Zinc stearate (90%, ZnSt_2_) was acquired from Riedel de Haën. Oleic acid (70%) came from Fisher Chemicals. All chemicals were used as received without any further purification, unless stated otherwise.

#### Zinc Ethylxanthate

Zinc ethylxanthate was prepared according to the procedure reported in Protiere and Reiss (2006).

#### Preparation of Indium-Myristate

Indium acetate (6.9 mmol), myristic acid (21.4 mmol), and ODE (15 mL) were mixed in a 50 mL three neck flask. The solution was stirred and degassed under vacuum for 3 h at 120°C. After cooling down to room temperature the precipitated indium-myristate was washed with ca. 150 mL of dry hexane before drying under vacuum.

#### Preparation of Zinc-Oleate

Zinc acetate (5 mmol), oleic acid (10 mmol), and 9.35 mL ODE were mixed in a 50 mL three neck flask. The solution was stirred and degassed under vacuum at 120°C for 1 h. After cooling down to room temperature the flask was backfilled with Ar and the mixture was stored in the glove box.

#### Preparation of TOP-Se and TOP-S

For a 0.4 M TOP-Se stock solution, 2 mmol of selenium powder were dissolved in 5 mL of trioctylphosphine (TOP) for 24 h. The preparation of a 0.4 M TOP-S stock solution was prepared similarly using elemental sulfur.

#### Synthesis of InZnP Core Nanocrystals

Indium-myristate (0.1 mmol), ZnSt_2_ (0.2 mmol), and 8.5 mL ODE were mixed in a 50 mL three-neck flask and degassed for 1 h. The flask was backfilled with Ar and the reaction mixture was rapidly heated to 300°C in a molten salt bath. At 100°C 0.1 mmol (TMS)_3_P, dissolved in 1 mL ODE, was swiftly injected. After 20 min the reaction was quenched by removing the salt bath and letting cool down to room temperature.

#### Synthesis of InZnP/ZnSe_x_S_1−x_ Core/Gradient Shell Nanocrystals

The InZnP core was prepared as mentioned before but instead of cooling down to room temperature the mixture was kept at 220°C. 2.5 mL of the 0.4 M zinc-oleate (1 mmol) stock solution was added dropwise followed by the swift injection of 0.444 mL of TOP-Se (0.2 mmol) stock solution mixed with 0.5 mL ODE and 1.57 mL of TOP-S (0.7 mmol) stock solution. The reaction mixture was heated to 300°C using a heating mantle with a heating ramp of 10°C per minute. The overall shell growth time was ca. 30 min and the reaction was quenched by cooling down to room temperature. The NCs were purified three times by a precipitation (1:1 v/v mixture of methanol/chloroform and acetone) and redispersion (chloroform) cycle and finally stored in hexane.

#### Synthesis of InZnP/ ZnSe_x_S_1−x_/ZnS Core/Shell/Shell Nanocrystals of Variable ZnS Shell Thickness

The synthesis of the InZnP/ZnSe_x_S_1−x_ NCs was performed as described above but instead of cooling down to room temperature the mixture was kept at a temperature of 230°C using a heating mantle. In order to prepare the second (thin) ZnS shell, a mixture of 0.1 mmol zinc ethylxanthate dissolved in 100 μL DMF and 1 mL toluene was added to 0.8 mmol ZnSt_2_ dissolved in 3 mL ODE and injected into the reaction mixture using a syringe pump with a pump rate of 8 mL/h. After 30 min of shell growth the reaction was quenched by cooling down to room temperature and the NCs purified as described above for the gradient shell. To grow a thick ZnS shell the amount of zinc ethylxanthate and zinc stearate solution was doubled and the shell growth time was extended to 60 min before quenching the reaction by cooling down to room temperature.

#### Synthesis of InZnP/ZnSe_x_S_1−x_/ZnS-Alumina

InZnP/ZnSe_x_S_1−x_ NCs synthesis was performed as described above and the mixture was kept at 230°C after gradient shell growth for 30 min. With a syringe pump, a shell mixture used for the preparation of a thin second ZnS shell was injected over the course of 30 min (0.1 mmol zinc ethylxanthate dissolved in 100 μL DMF and 1 mL toluene was mixed with 0.8 mmol ZnSt_2_ dissolved in 3 mL ODE). When the injection of shell mixture started, 1 mL of a solution prepared by dissolving 1.12 mmol Al (IPA)_3_ in 3 mL ODE at 40°C was slowly injected. At 10 and 20 min of the shell growth another 1-mL volume of the Al-mixture was injected. The mixture was kept for a total of 60 min at 230°C before cooling down to room temperature and purifying as mentioned before.

#### Embedding InP-Based QDs in PMMA

To prepare a 30wt % PMMA solution, 3 g of PMMA were dissolved in 7 mL chloroform, stirred for 1 h and ultrasonicated several times in between for 2 min. The QD solutions had a concentration of approximately of 20 mg/mL and in order to prepare a PMMA disc with 0.4 wt% (~10^−6^ mol^.^L^−1^), ca. 12 mg (620 μL) InP-based NCs were added to the PMMA solution. The mixture was stirred for another hour and ultrasonicated in between until the QDs were homogenously distributed. 1.5 mL of the QD-PMMA solution was filled in a Teflon mold (diameter 20 mm) and kept for 3 days in the dark to enable the slow evaporation of chloroform. The dried PMMA discs were checked to ensure the absence of any bubbles, which can appear in case the chloroform evaporates too fast. They were then unmolded and cut using a gauge in order to have the same geometry and volume for all samples (disc diameter: 15 mm, thickness: 1 mm).

#### Phase Transfer Using Penicillamine

The InZnP QDs with different shells (except for alumina coating) were rendered water-soluble via ligand exchange using D-penicillamine. Therefore, a 0.2 M solution of D-penicillamine was prepared in 1 mL of degassed MilliQ water. After addition of 200 μL TCEP the pH was adjusted to 9 with TMAOH (25 wt% in methanol) solution. After thorough degassing, 0.5 mL of the ligand solution was added to 1 mL degassed colloidal solution of QDs dissolved in chloroform with a concentration of ca. 3–5 μM. The biphasic mixture was stirred vigorously for 45 min at room temperature. The mixture was quickly centrifuged to obtain a clear phase separation. The upper layer containing the QDs in the aqueous phase was separated from the organic phase and purified from excess ligands using a NAP^TM^10 size exclusion column (Sephadex^TM^ G-25 DNA Grade from GE Healthcare). The column was equilibrated with a 1X PBS buffer, which was further used as eluent and storage buffer. The transferred QD samples were stored at 4°C.

### Characterization

#### Photophysical Characterization

Absorbance measurements were performed using a Hewlett Packard 8452A spectrometer and the emission spectra were recorded using a Fluorolog FL3-22 spectrometer from Horiba-Jobin Yvon equipped with a 150 W xenon lamp for steady-state measurements and a NanoLED laser diode from Horiba with a wavelength of 350 nm and 1 MHz repetition for time-resolved measurements. Decay curves were fitted using Decay Analysis software from Horiba Scientist and intensity averaged decay times calculated according to Equation 1.

(1) $$\begin{array}{r} \tau <int>=\frac{\sum_{i}A_{i}^{*}\tau_{i}^{2}}{\sum_{i}A_{i}^{*}\tau_{i}} \end{array}$$

In Equation 1 A_i_ is the amplitude of the respective decay time t_i_. Photoluminescence quantum yield measurements were performed at room temperature using an integration sphere, Hamamatsu Quantaurus Absolute PL quantum yield spectrometer.

#### Structural Characterization

Powder X-ray diffraction was performed using a Panalytical X'Pert diffractometer equipped with copper anode (λK_α1_ = 1.5406 A, λK_α2_ = 1.5444 A) and an X'Celerator 1D detector. The elemental composition was analyzed using a Zeiss Ultra 55+ scanning electron microscope equipped with an EDX probe. The XRD and EDX samples were prepared by drop-casting a concentrated NCs dispersion either on a disoriented silicon substrate or on a cleaned silicon substrate. Transmission electron microscopy (TEM) was performed at cryogenic temperature on a FEI Polara microscope operating at 300 kV and images were recorded on a Gatan K2 camera. For sample preparation, around 4 μL of a diluted QD dispersion was applied onto a 400-mesh copper TEM grid covered with a homemade carbon film and the solvent was allowed to evaporate.

#### XPS Studies

Chemical characterization by XPS was carried out using a Versaprobe II ULVAC-PHI spectrometer. A monochromatic beam (X-ray source Al-K_α_ 1486.6 eV) of 100 μm diameter and 97 W power was focused on the surface of the samples. Survey spectra were measured over a spectral range of 0–1200 eV to identify the elements present in the material using a pass energy of 117 eV which corresponds to a resolution of 1.6 eV. High-resolution spectral analyses were performed using a pass energy of 23 eV which corresponds to a resolution of 0.5 eV. All XPS measurements were carried out in an ultra-high vacuum chamber (7.10^−8^ mbar).

All XPS spectra binding energies were corrected using the C 1s line of alkyl groups of PMMA at 285.0 eV. Curve fitting and background subtraction were accomplished using Casa XPS software. The spectra curve fitting was performed using Voigt function, convolution product of Gaussian (80%) and Lorentzian (20%) distributions.

#### Aging Tests in a Climatic Chamber

The QD-PMMA disks were placed in a Q-SUN Xe-1 Xenon arc test chamber (Q-LAB) providing full sunlight spectrum and continuously irradiated up to 32 h. Weathering conditions were adapted from the ISO norm 4892-2:2006, developed for studying the aging of plastics. The irradiance and temperature were fixed at 1.44 W/m^2^ (measured at 420 nm) and 40°C, respectively, with no humidity control. PL measurements were performed after an irradiance time of 6, 12, 24, and 32 h.

#### Toxicity Testing

Human skin samples were obtained following breast surgery from healthy female donors with their informed consent (Centre Hospitalier Universitaire de Grenoble, Grenoble, France). Keratinocytes were isolated as previously described (Mouret et al., 2006). They were grown at 37°C, 5% CO_2_, in keratinocyte serum-free medium (KSF-M) supplemented with 1.5 ng/ml EGF, 25 μg/ml bovine pituitary extract, 75 μg/ml primocin. For all experiments, cells were used at passages 1 or 2. They were seeded in 96-well plates at 5,000 cells per well. When they reached 60% of confluence, they were exposed for 24 h to 6.25–100 nM of QDs. Exposure medium was then sampled and used to assess the release of lactate dehydrogenase (LDH) from cells (LDH assay kit, Sigma-Aldrich), which is a marker of cell membrane integrity, i.e., cell viability. The ability of these cells to proliferate was evaluated by quantifying the incorporation of 5-Bromo-2'-deoxy-uridine (BrdU) in their DNA (BrdU assay kit, Roche). Both assays were performed following manufacturer's instructions. Triton X100 (0.1%) was used as positive control.

## Results and Discussion

### Characterization of the Different Core/Shell Systems

**Figure 2:**
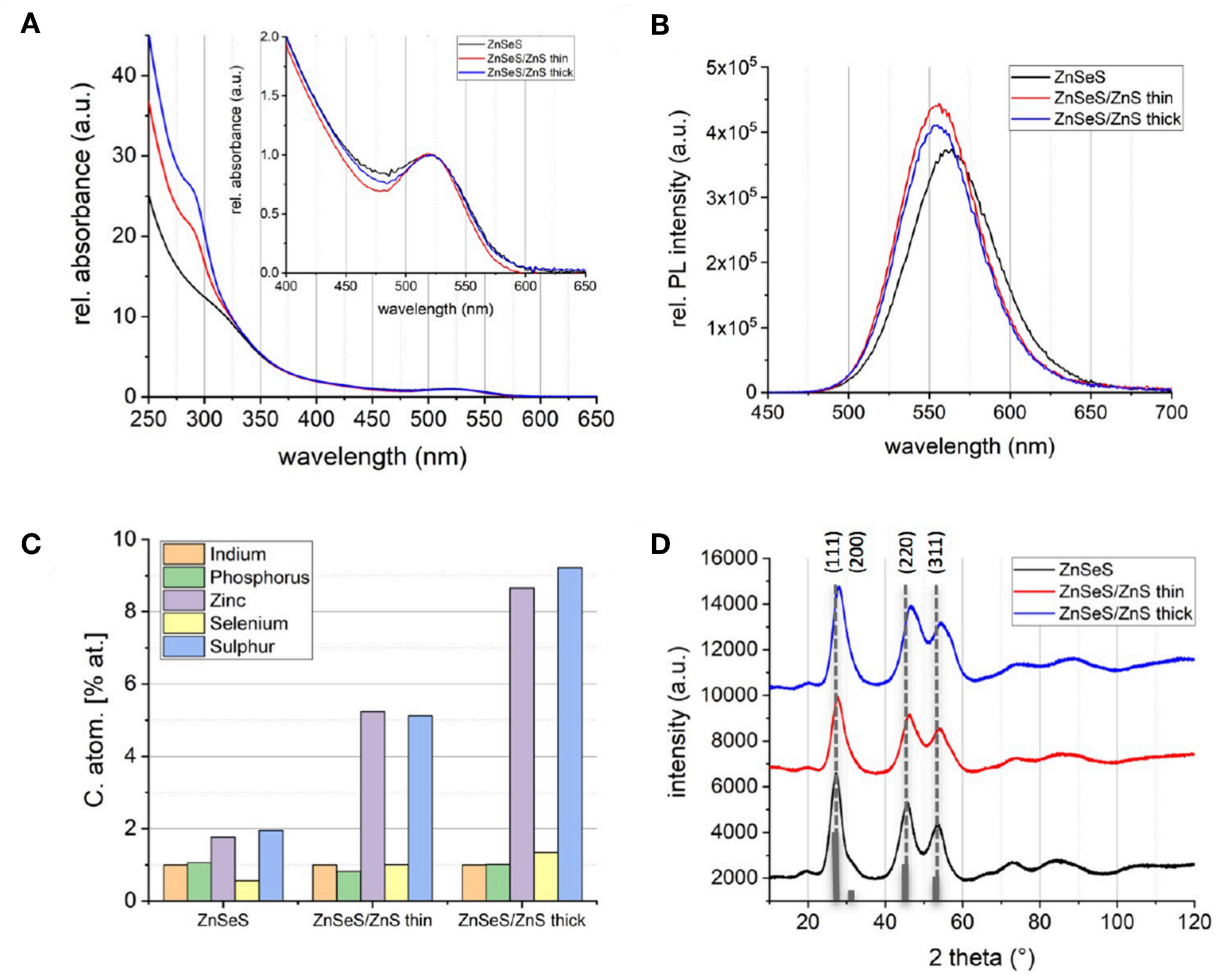
Normalized absorbance spectra **(A)** and absorbance corrected PL spectra **(B)** of InZnP QDs with ZnSeS (black), ZnSeS/ZnS thin (red), and ZnSeS/ZnS thick shell (blue). **(C)** Indium normalized composition determined with EDX. **(D)** Powder X-ray diffractograms (Cu-Kα); the reference pattern of bulk zinc blende InP (JCPDS file #32-0452) is added as gray bars.

Figure 2 shows the UV-vis absorption and photoluminescence (PL) spectra as well as EDX data of InZnP/ZnSe_x_S_1−x_ core/gradient shell QDs before and after growth of an additional ZnS shell. In contrast to literature methods, the monomolecular precursor zinc ethylxanthate has been applied for ZnS growth. As expected, the absorption spectra show an enhanced absorbance below 350 nm with increasing ZnS shell thickness. The EDX data (Figure 2C) also confirms unambiguously the successful growth of the thin and thick ZnS shells on the core/gradient shell system (**Figures 4C,D**). The same core diameter (2.67 ± 0.32 nm from TEM measurements) was used for all samples. In accordance with the elemental increase determined from EDX and TEM data, the thicknesses of the shells are around 2 monolayers (gradient shell), 3 monolayers (thin ZnS shell) and 4–5 monolayers (thick ZnS shell). Due to the fact that the gradient shell already provides a very good surface passivation, the additional ZnS growth only slightly improves the PL intensity (PLQY: 50.9% gradient shell, 56.5% CSS thin, 63.6% CSS thick). A small blue shift of the PL maximum with increasing shell thickness (cf. Table S1, up to 9 nm for the thick ZnS shell) likely originates from the strain exerted on the core due to the smaller lattice parameter of ZnS. This hypothesis is confirmed by powder X-ray analyses, showing a shift of the diffraction peaks characteristic of InZnP to wider angles upon ZnS shell growth (cf. Figure 2D). Time-resolved PL studies (Figure S1, Table S2) reveal similar averaged decay times on the order of 65–67 ns before and after ZnS shell growth, however, the shortest lifetime is constantly decreasing with increasing ZnS shell thickness, suggesting a smaller contribution of surface trap states (Wegner et al., 2019).

As an additional surface passivation step, coating with an alumina layer was explored. While this treatment has been shown to yield a significant stability enhancement of CdSe and CuInS_2_-based NCs (Li et al., 2015; Yan et al., 2016), it has not yet been applied to indium phosphide based QDs. To induce the formation of the alumina coating, we added a well-defined amount of aluminum isopropoxide during the growth of the (thin) ZnS shell on the gradient shell (cf. Experimental Section). While the absorption and PL spectra remain essentially unchanged with respect to the CSS system (Figure S2) and a high PL QY is maintained (48%), aluminum treatment induces a higher value of the averaged PL decay time (82.8 ns, cf. Table S3). EDX analysis shows that a significant amount of aluminum has been incorporated into the QDs with an In:Al ratio of 1:9. Such a high ratio is indeed required for forming, as hypothesized, a monolayer of alumina on an In(Zn)P core already passivated with a gradient and a thin ZnS shell.

### Photostability in PMMA

**Figure 3:**
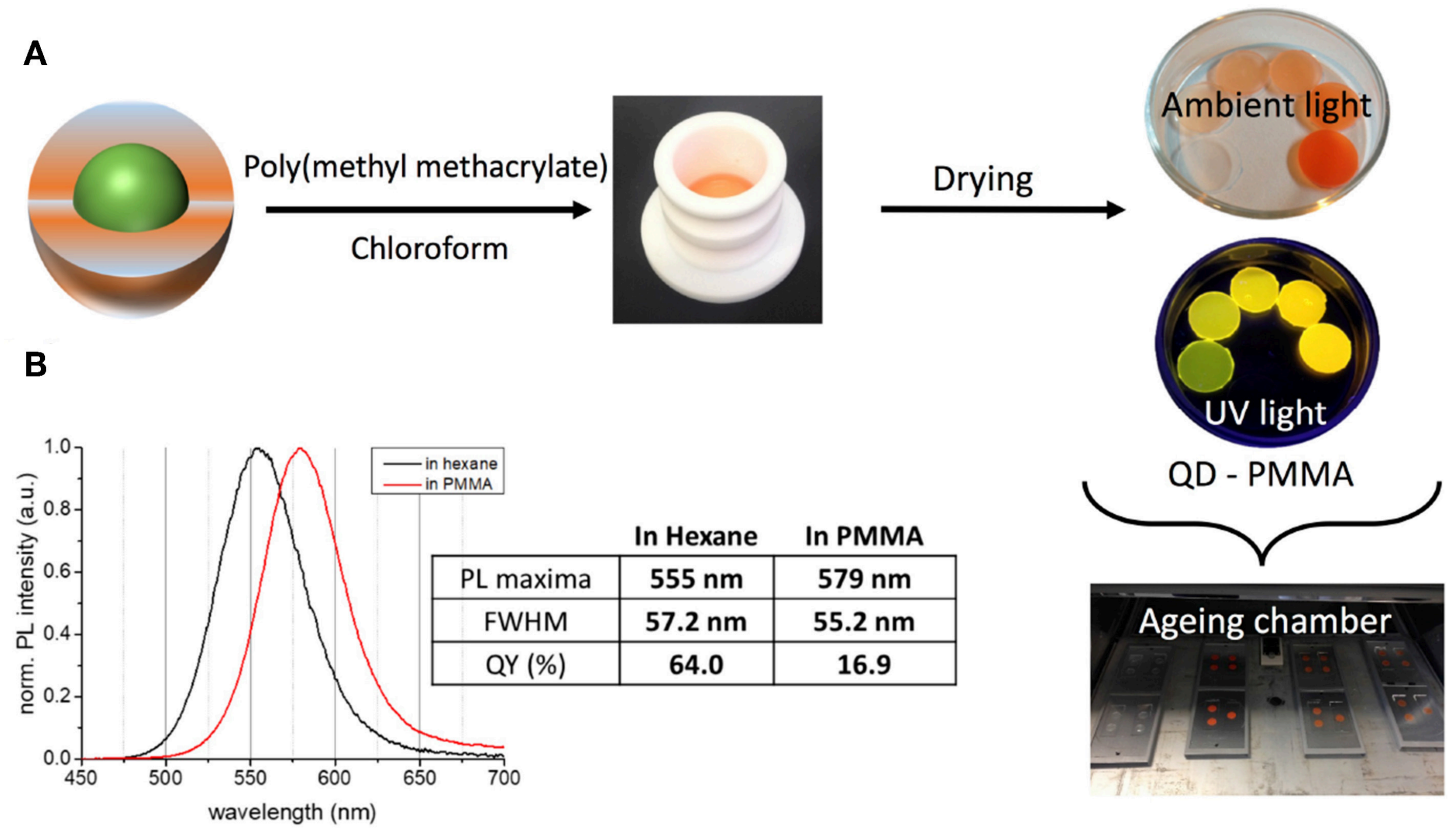
**(A)** Processing of the QDs for aging experiments using a PMMA matrix and different concentrations of QDs. **(B)** Comparison of the PL properties of InZnP/ZnSeS/ZnS (thick shell) QDs in colloidal solution (hexane) and after embedding in the PMMA matrix.

For assessing the photostability under controlled conditions, the QDs were first integrated into a PMMA matrix. The main advantages of PMMA are its optical transparency (Figure S3), stability under irradiation, amorphous character, ease of composites processing, and the mechanical stability of the obtained monolithic materials. Figure 3 depicts the different steps of composite preparation and aging experiments. By optimizing the PMMA concentration in chloroform (30 wt%) and slow drying, homogeneous composites without air-bubbles could be obtained, suitable for optical measurements. A QD loading of 0.4 wt% resulted in the best compromise of film homogeneity and PL QY (cf. Table S4).

Incorporation into the PMMA matrix leads to a significant bathochromic shift of the PL emission maximum with increasing QD concentration in the composite (cf. Figure 3, Figure S4), accompanied by a small reduction of the PL line width. For the gradient shell and CSS system this shift accounts for 26 and 24 nm (97 meV/93 meV), respectively, while it is less pronounced in the case of the aluminum treated sample (14 nm, 56 meV). At the same time, a significant decrease of the PLQY is observed with the retained QY following the order CSS (16.9%, decrease: 73%) > aluminum treated (10.1%, decrease: 79%) > gradient shell (7.6%, decrease: 85%). Similar effects have been observed in other studies on QD incorporation into a PMMA matrix via physical mixing of both components (Zvaigzne et al., 2015). We attribute both phenomena (PL shift and QY decrease) to the fact that in the thick QD-polymer composite radiative or non-radiative energy or charge transfer processes take place favored by the close proximity of the QDs and their size distribution.

As already studied in close-packed InP-based QD films without polymer matrix (Ung et al., 2010a), in principle three processes can occur: (i) resonance photon reabsorption; (ii) Förster resonance energy transfer; (iii) direct excited charge carrier transfer from one QD to another. Due to the expected increased spatial separation of the QDs in the polymer as compared to close-packed films, process (i) could be favored here. However, it has been shown in many literature examples that the physical mixing of QDs with the polymer matrix can induce phase segregation and aggregation of the QDs (Ghimire et al., 2018). Therefore, processes (ii) and (iii) can also take place in the present case. The identification of the predominant mechanisms requires additional studies which go beyond the scope of the present work.

**Figure 4:**
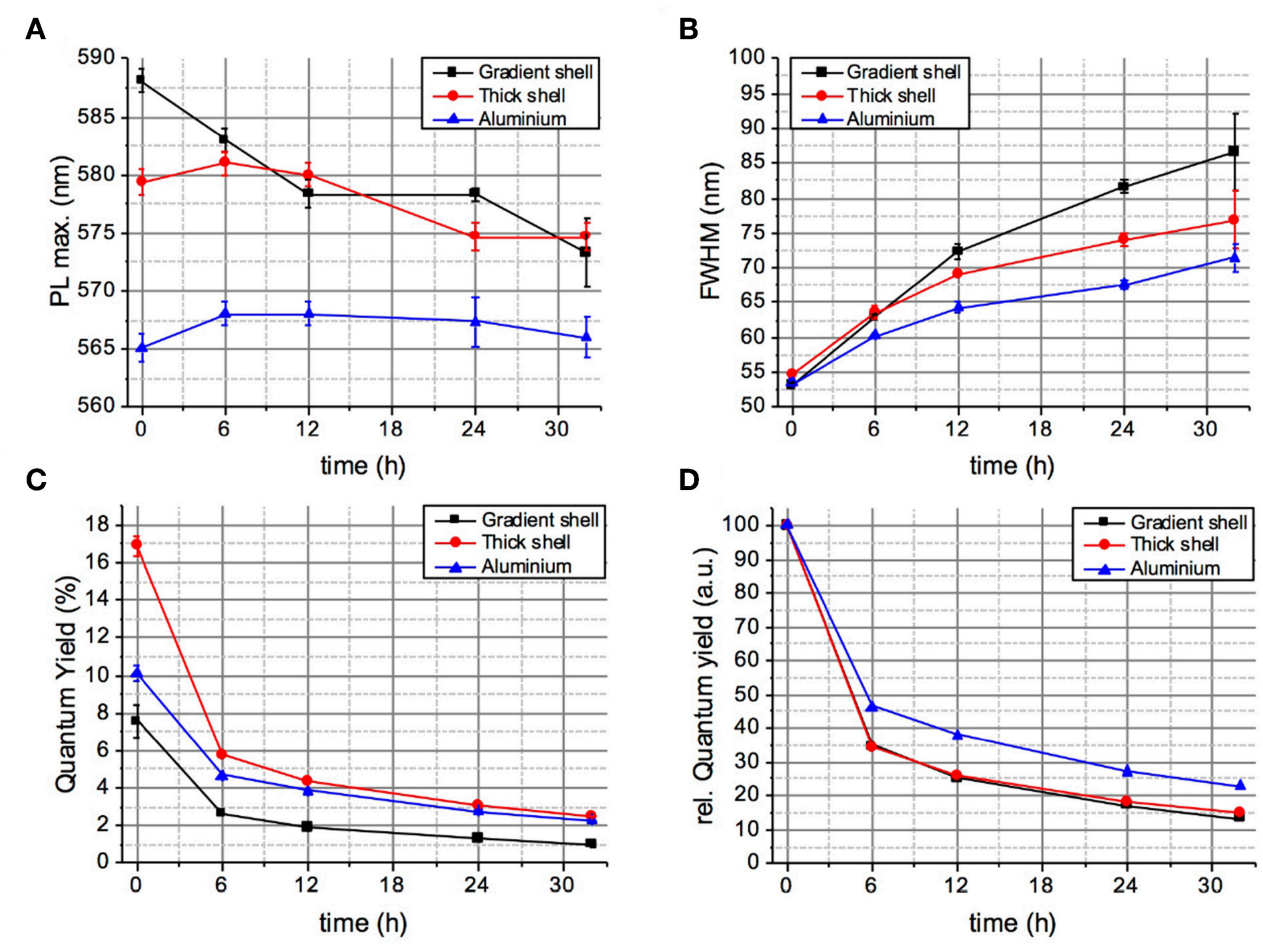
Evolution of the PL maximum wavelength **(A)**, linewidth **(B)**, absolute PLQY **(C)**, and relative PLQY **(D)** of the QD-PMMA composites during 32 h of continuous aging (irradiation with a solar simulator at 40°C).

During 32 h of continuous irradiation with simulated solar light in an aging chamber at 40°C, the gradient shell system exhibits a hypsochromic shift of the PL maximum of 55 meV, while the position of the other two samples is essentially stable (Figure 4A). Nonetheless, all samples show an increase of the emission linewidth, which is most pronounced for the gradient shell, followed by the thick shell and the aluminum treated sample (Figure 4B). The evolution of the PLQY confirms this last sample to be the most photostable one: as visible from the comparison of the relative QY loss under irradiation (Figure 4D), the gradient shell and CSS systems present identical behavior, while the aluminum treated sample maintains a higher fraction of the initial PL intensity. Intriguingly, all studied samples exhibit a very similar degradation kinetics displaying a fast PL loss during the first 6 h of irradiation before reaching a regime exhibiting a much slower slope and eventually a plateau for extended durations. To confirm the absence of influences arising from potential aging effects of the PMMA matrix the latter has been submitted to identical irradiation experiments on its own (i.e., without QDs) (Figure S5).

Noteworthy, the general trends of PL intensity evolution of the three systems are similar when keeping the composites at room temperature in the dark (cf. Figure S6), while no evolution of the optical properties is observed for samples kept in the colloidal state either in organic solvent or in PBS buffer after aqueous phase transfer (vide infra). In the PMMA matrix, the QY gradually decreases over time for the CSS and gradient shell systems, while it remains stable for the aluminum treated sample. In contrast to the case of solar irradiation, no fast QY loss during the first hours is observed, which clearly confirms that this fast degradation is light induced. As visible from the evolution of the normalized PL spectra with irradiation duration (Figure S7), in all three samples the contribution of trap-state emission in the 650–750 nm range markedly increases over time. One possible explanation for the activation of trap states and the observed PL decrease in the first 6 h of irradiation is light-induced rearrangement of the nanocrystal surface (Saba et al., 2013). This process, leading ultimately to photocharging and charge trapping, is influenced in the present case by interaction of the QD surface with the polymer matrix.

### XPS Analysis

**Figure 5:**
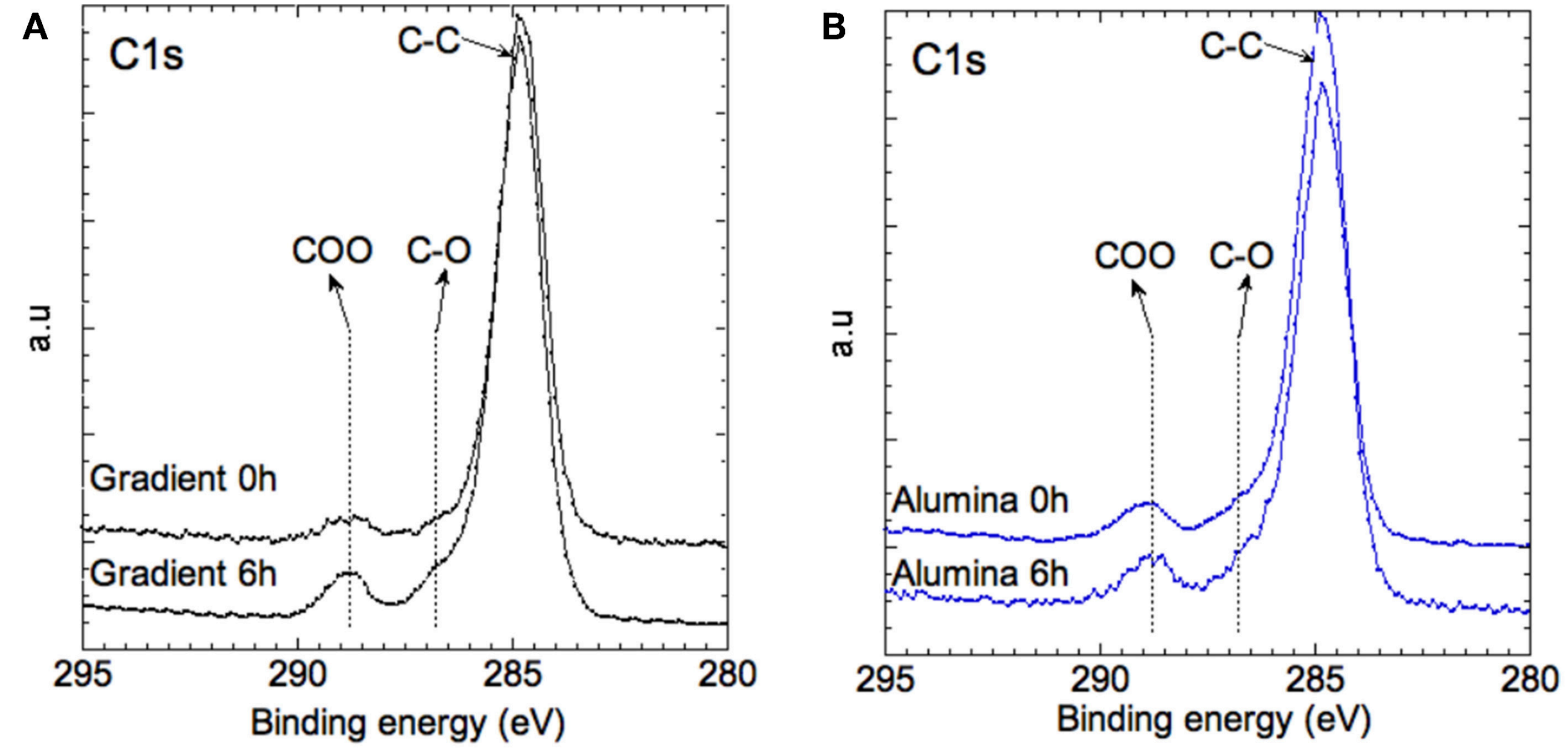
XPS spectra of the C1s core level of **(A)** the gradient shell sample and **(B)** the aluminum treated sample at 0 and 6 h of aging.

To probe the change of surface chemistry of QDs as well as PMMA stability over aging, we performed XPS analyses for which the probed depth is about 5 nm. Two samples were studied before and after 6 h of irradiation: InZnP/ZnSeS gradient shell QDs, most affected by the aging, and aluminum treated CSS QDs, least affected, both embedded in the PMMA matrix. Figure 5 shows the C1s core level of the pristine and irradiated samples. The main feature of the spectra is that the signatures of PMMA are preserved after 6 h of irradiation, for instance the C-C (285.0 eV), C-O (286.7 eV), and COO (288.7 eV) signals, indicating that PMMA remains stable during the selected aging time.

**Figure 6:**
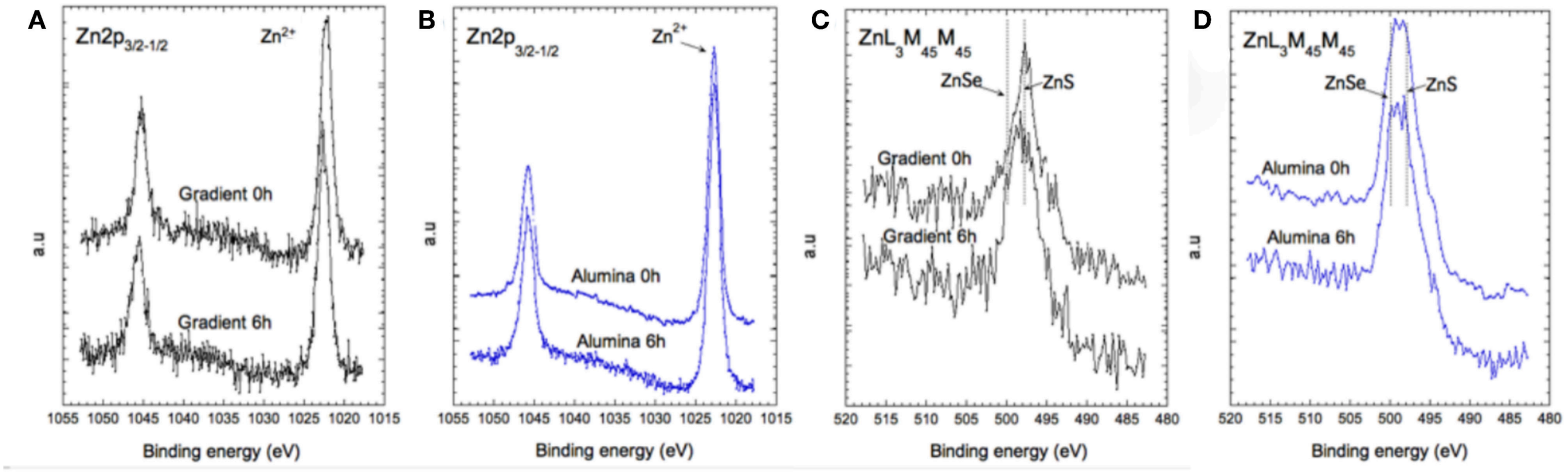
XPS spectra of the Zn 2p core level **(A,B)** and of the Auger transition Zn L_3_M_45_M_45_ **(C,D)** for the gradient shell sample and the aluminum treated sample at 0 and at 6 h of aging.

The valence change of zinc was studied through the analyses of the Zn 2p core peak and Zn L_3_M_45_M_45_ Auger lines (Figure 6). The Zn 2p signal shows a main peak at 1022.7 eV assigned to the +II valence state of zinc. The binding energy of Zn 2p_3/2_ does not change during aging in the case of the aluminum treated sample, while a slight shift (+0.3 eV) to higher binding energies is observed for the gradient QDs after 6 h of irradiation. To further investigate the chemical environment of the zinc atoms, analysis of the Zn L_3_M_45_M_45_ Auger peaks provides detailed information, which can be extracted from the shapes, intensities, and locations of the Auger lines involving deep Zn 2p_3/2_ (L_3_) and shallow Zn 3d_5/2−3/2_ (M_45_) core levels. The Auger line arises from the three nearly degenerated levels 1D, 3P, and 1G, and two extreme levels 1S and 3F constituting the higher and lower energy shoulders of the Auger line (inset of Figure 6). As a first approximation, the main peak of each chemical group can be represented by the 1D, 3P, and 1G triad (nX) symbolized by a dashed line (Lee et al., 2014). With these information, the Wagner parameter can be defined (Wagner, 1972):

$$\begin{array}{l} \alpha =E_{\text{b}}\left(\text{Zn}2{\text{p}}_{3/2}\right)+{\text{E}}_{\text{K}}\left(\text{Zn LMM}\right) \end{array}$$

where E_k_ is the kinetic energy of the core–valence–valence Auger transition electron, and E_b_ the binding energy of the Zn2p_3/2_ core electron referred to the Fermi energy.

Materials with similar Wagner parameters have closely similar extra-atomic relaxation arising from multiplet coupling in the final states, i.e., the extra-atomic polarization energy and the local electronic structure of the material. Hence, we can distinguish at least three families of zinc-ligand species for both types of samples (gradient shell and aluminum treated), since we can extract at least three Wagner parameters: α_1_ at 2009.6 eV, α_2_ at 2011.1 eV and α_3_ at 2013.1 eV. They can be assigned to zinc in ZnSe, ZnS, and Zn-Zn chemical environment, respectively. At this stage, the contribution of the ZnSe and ZnS phases is difficult to quantify from the spectral signature. However, it appears that in the case of the aluminum treated QDs the zinc related environment remains stable over time. In the case of the gradient shell QDs, the ZnS related bonds are dominant in the pristine material and decrease after 6 h of aging, while the ZnLMM spectral shape becomes similar to that recorded in the case of aluminum treated QDs.

**Figure 7:**
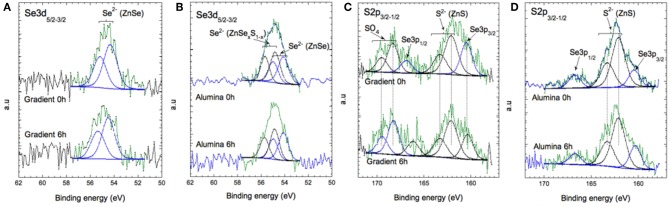
XPS spectra of Se 3d **(A,B)** and of S 2p and Se 3p **(C,D)** for the gradient shell sample and the aluminum treated sample at 0 and 6 h of aging.

Figures 7A,B reports the Se 3d core level of both systems. In the case of the gradient shell, the spectra show one doublet at 54.1–55.0 eV (associated to spin-orbit coupling), signature of Se^2−^ ions in ZnSe. In the case of the aluminum treated QDs, we observe two doublets at 54.1–55.0 and 54.9–55.8 eV associated with ZnSe and ZnSe_x_S_(1−*x*)_, respectively. These differences are unexpected, as the core and gradient shell were synthesized in the same manner for both systems. On the other hand, as the additional growth steps in the case of the alumina-capped sample imply prolonged heating to 230°C, a more pronounced interdiffusion of the ZnSe and ZnS phases may take place giving rise to the mixed ZnSe_x_S_(1−*x*)_ phase. The S 2p doublet (Figure 7D) at 162–164 eV is the signature of S^2−^ ions in ZnS environment. The ZnS:ZnSe ratio remains stable during aging (cf. Table S5). For the gradient shell, the presence of oxidized sulfur (–SO_4_) is observed (Figure 7C) whose concentration increases slightly after aging.

**Figure 8:**
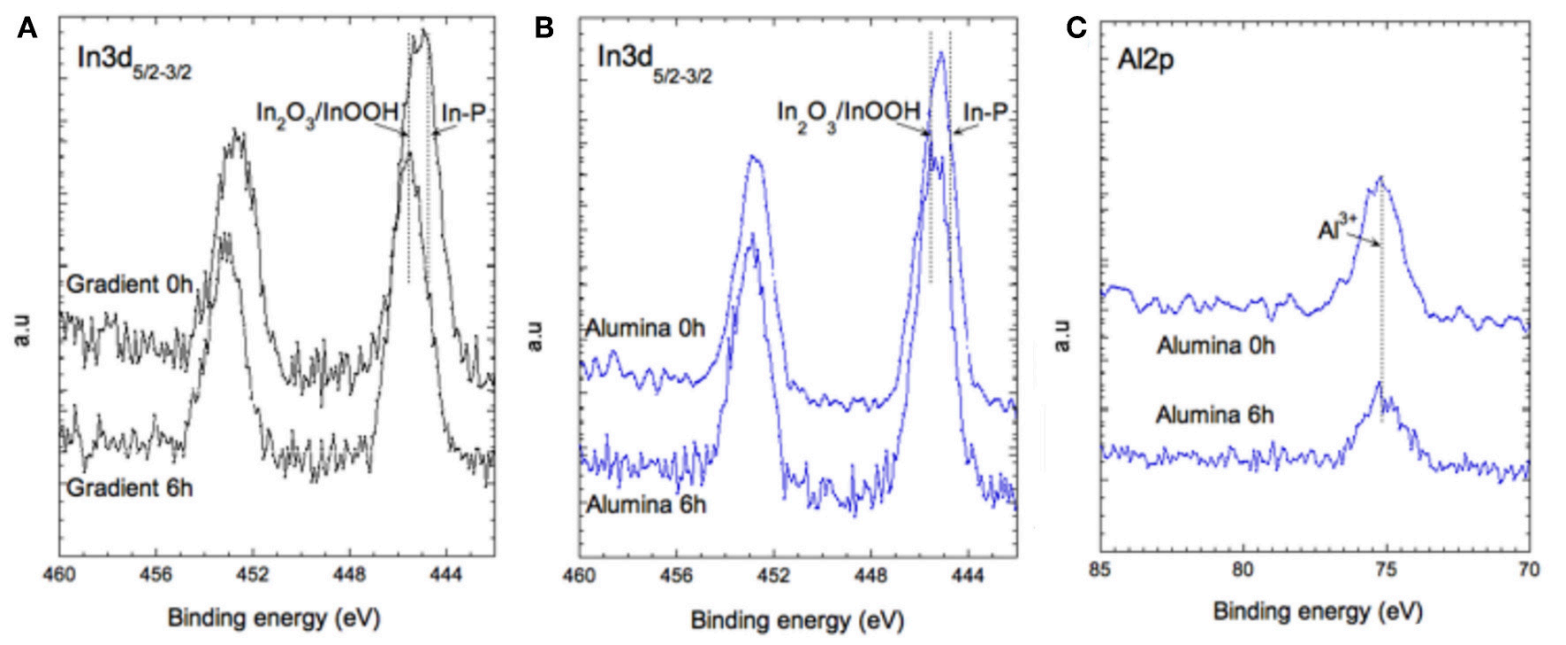
XPS spectra of In 3d for **(A)** the gradient shell sample and **(B)** the aluminum treated sample at 0 and 6 h of aging. **(C)** XPS spectra of Al 2p for the aluminum treated sample.

At a first approximation, the In 3d_5/2_ peak recorded at the surface centered at 445.4 eV can be assigned to In^3+^ ions in In_2_O_3_ and/or In (OH)_3_. The presence of In (S, Se, P) cannot be excluded since the P 2p core level peak (not shown) exhibits two contributions, a major one around 134 eV attributed to phosphate groups (InPOx) and a low intensity peak around 130 eV assigned to In-P bonds (Figure 8). Oxidation of the core/shell interface in InP-based QDs occurring during the synthesis under similar experimental conditions has been reported by Delpech and coworkers (Virieux et al., 2012). It has been attributed to the *in situ* generation of minute amounts of water due to side reactions of the fatty acid ligands (e.g., myristate, stearate).

In the case of the aluminum treated sample, the ratio between both indium species remains quasi-stable whereas in the case of the gradient system significate changes are observed (Figures 8A,B).

The Al 2p core level is the signature of Al^3+^ like in Al_2_O_3_. It stays the same after 6 h of aging, confirming that the aluminum coating is very stable and does not degrade under irradiation (Figure 8C). Taken together, the presented XPS results fully underpin the stability differences of the two systems expected from the photophysical studies. In the case of the gradient shell, the initial ZnS outer layer already shows distinct signs of oxidation right from the start (–SO_4_ species), which are not detectable in the aluminum treated sample. Furthermore, during the aging process the degradation of the gradient shell sample continues, as visible by the XPS spectral changes, in particular the decrease of the signals characteristic for ZnS related bonds and increase of the oxidized indium component. The latter implies a reduction of the emissive core size, which is supposed to be at the origin of the observed hypsochromic shift of the PL maximum (cf. Figure 4A).

### Cytotoxicity Studies

**Figure 9:**
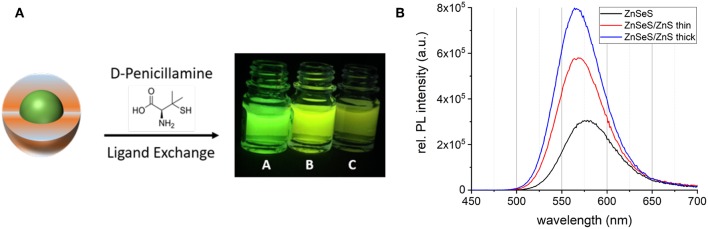
**(A)** Schematic representation of the aqueous phase transfer using penicillamine. The photograph shows the phase transferred samples: InZnP/ZnSeS/ZnS thick (A), InZnP/ZnSeS/ZnS thin (B), and InZnP/ZnSeS (C) in PBS buffer. **(B)** Absorbance corrected PL spectra of the phase-transferred samples.

Aqueous phase transfer of the QDs was achieved by surface ligand exchange with zwitterionic penicillamine (Figure 9) (Tamang et al., 2011; Mattera et al., 2016). In this procedure, the QDs in chloroform are brought into intimate contact with an aqueous solution of penicillamine. The pH of the aqueous phase is adjusted in the basic range (around 9), to favor the deprotonation of the thiol moiety leading to efficient binding to the ZnS surface of the QDs. In the case of the alumina-coated system, however, this approach was not successful. Other types of surface chemistries have to be developed there, which are the subject of ongoing research. Therefore, in the remaining part of this article we will focus on the core/shell systems without aluminum treatment.

While the absorbance properties are not affected by the phase transfer, the PL peaks exhibit a bathochromic shift of around 10–15 nm accompanied by a slight line broadening (Table S6). The PL intensity strongly depends on the shell thickness of the QDs as seen in the photograph in Figure 9A and in the spectra in Figure 9B. As generally observed in phase transfer methods applying direct surface ligand exchange, the PLQY significantly decreases. This decrease is particularly pronounced for the gradient shell sample (QY: 6.0%) whereas the systems with additional ZnS shells exhibit enhanced PL conservation (QY: 15.5% CSS thin; 17.1% CSS thick). The gradient shell sample shows a significant decrease in the average decay time after phase transfer and a concomitant increase of the amplitude of the shortest lifetime (Figure S8, Table S7). All three systems exhibit an increase of the latter, albeit the trend of the contribution of the short lifetime component is maintained in the order gradient shell > CSS thin > CSS thick, as in the samples before phase transfer. All in all, these results indicate that surface trap states induced by the ligand exchange reaction are at the origin of the PLQY decrease. As expected this detrimental effect can be reduced by increasing the shell thickness. Dynamic light scattering (DLS) measurements (Figure S9) confirm the absence of larger-sized aggregates. The hydrodynamic diameters of the samples extracted from DLS are consistent with the values obtained from TEM analysis (Figure S10). However, the precise determination of the QD sizes is challenging due to the low contrast of the ZnS shell in TEM and the small diameters in the 4–6 nm range.

**Figure 10:**
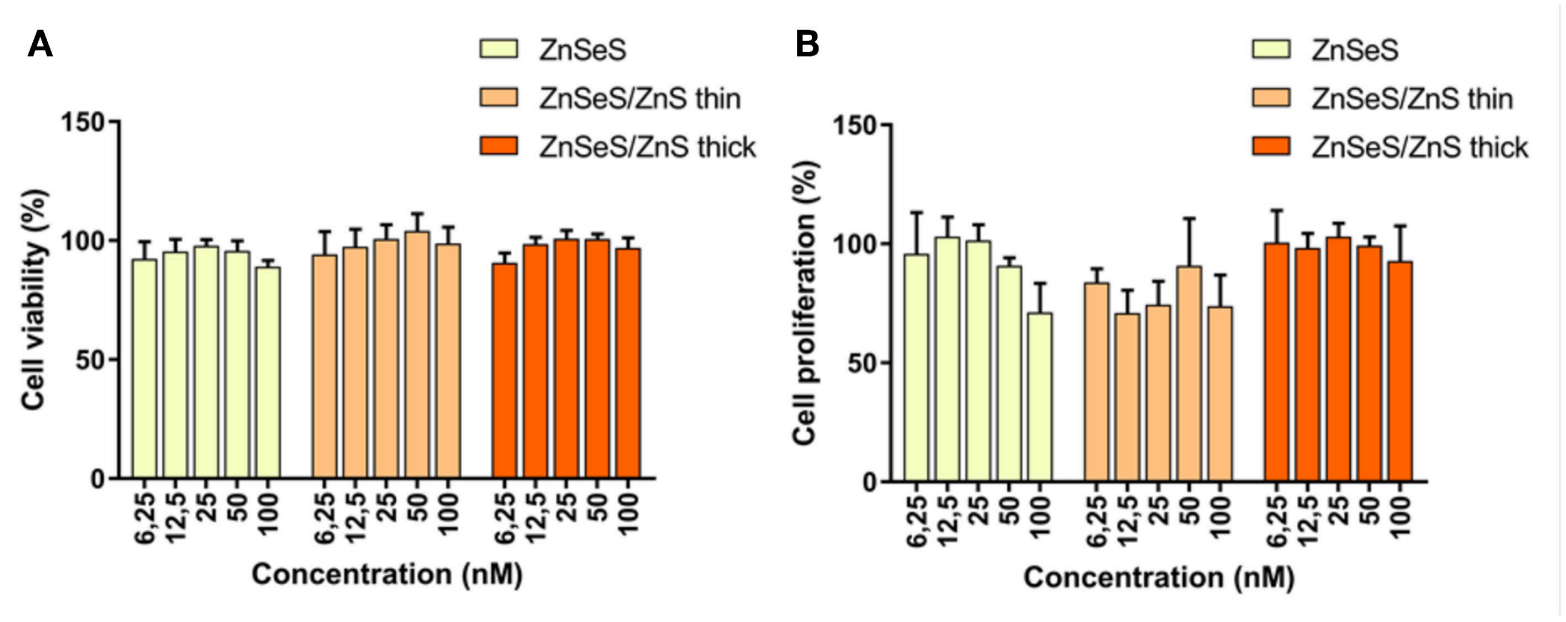
Cell viability **(A)** and cell proliferation **(B)** of human keratinocytes exposed to increasing concentrations of QDs with different shells.

Since the QDs are synthesized in liquid phase with no risk of aerosolisation, the most probable exposure route for humans is through skin contact (Reiss et al., 2016). Impact of these QDs was assessed *in vitro* on skin cells, i.e., primary keratinocytes isolated from human donors. As shown in Figure 10A, exposure to 6.5–100 nM of QDs for 24 h do not cause any leakage of lactate dehydrogenase from cells, i.e., do not affect cell membrane integrity, which is classically used as a proxy for cell viability. BrdU, an analog of the nucleoside thymidine which is incorporated into DNA during replication, was used as an indicator of QD impact on cell proliferation. A trend toward reduced cell proliferation is observed in cells exposed to QDs (Figure 10B). This trend is observed in cells exposed to QDs with a gradient shell and CSS with a thin shell, but not to CSS with a thick shell. This shows that the thick shell not only facilitates the phase transfer and preserves best the photophysical properties but is also beneficial from a cytotoxicity point of view. These features combined with the compact size of the penicillamine-capped CSS QDs make them particularly interesting for further applications in biosensing.

## Conclusions

With the goal to improve the photostability of InP-based QDs, we have synthesized three different types of core/shell structures. The first one uses a ZnSe_x_S_1−x_ gradient shell as reported by Lim et al. (2011). In the second, CSS system, additional growth of a ZnS shell of controlled thickness on top of the gradient shell has been undertaken, using the monomolecular precursor zinc ethylxanthate (Protiere and Reiss, 2006). Finally, in the third system alumina coating was performed on top of the ZnS shell. When incorporated into a PMMA matrix and submitted to continuous irradiation in a climate chamber, the three systems exhibit distinctly different behavior. While all of them show a marked drop of QY within the first 6 h of irradiation, the aluminum treated sample retains most of the initial PL intensity. The gradient shell and CSS systems show a minor but continuous loss of QY also in the dark, which is attributed to the interaction of the PMMA matrix with the Zn-based shell. It has been shown that PMMA-type polymers can act as efficient sorbents for Zn (II) ion removal (Jakóbik-Kolon et al., 2017). To the contrary, the aluminum treated sample is fully stable in the dark.

XPS studies revealed that upon irradiation the degradation of the ZnS outer layer and oxidation of the In(Zn)P core are at the origin of the observed decrease of PL intensity and hypsochromic shift of the PL maximum. XPS also confirmed the formation of an Al_2_O_3_-type layer in the case of the aluminum treated sample, which effectively protects against degradation. The chemical stability of the alumina capped sample being thus demonstrated, the observed decrease of PLQY upon continuous irradiation is ascribed to light-induced surface reconstruction. The modification of the configuration of surface atoms and capping ligands can introduce defect states favoring photocharging of the QDs and charge carrier trapping. Similar processes are also at work in the case of the gradient and CSS systems, in addition to photo-oxidation. These detrimental effects could likely be overcome by increasing the inorganic shell thickness, providing a more efficient separation of the emissive core and the environment.

With the gradient shell and CSS systems phase transfer was achieved using surface ligand exchange with penicillamine and a clear correlation between the retained PLQY and shell thickness was observed. Cytotoxicity studies indicate that the different types of QDs do not influence the cell viability of human keratinocytes. On the other hand, a trend of reduced cell proliferation is observed for cells exposed to higher concentrations of gradient shell and CSS QDs with a thin ZnS outer shell. This trend is not visible for the CSS QDs with a thicker ZnS shell, confirming both the necessity and advantageous features of thicker shells protecting InP-based QDs. Ongoing studies concern the aqueous phase transfer of alumina-coated QDs, either via encapsulation with ambipolar macromolecules (e.g., phospholipids) or through the identification of appropriate bifunctional molecules for ligand exchange. Furthermore, with the goal to reduce PL losses during the QD incorporation in the matrix, different types of suitable polymers (e.g., polystyrene, polydimethylsiloxane, or block copolymers) deserve attention as well as approaches preventing from undesired phase-segregation of the QDs and the polymer by introducing specific interactions between both components (Ghimire et al., 2018).

Concluding, the presented results contribute to the better understanding of the degradation mechanisms of core/shell QDs and to the development of InP-based QD heterostructures combining enhanced chemical and photostability, bringing them closer to the requirements of real-life applications.

## Data Availability

All datasets generated for this study are included in the manuscript and/or the Supplementary Files.

## Author Contributions

MC and PR conceived and designed the study. KW performed the synthetic work and data analysis with assistance of PR and MC. AB performed the XPS studies and analysis. DT-B carried out the aging experiments and assisted in the XPS analysis. FD and DB performed cytotoxicity studies. LM helped with QD phase transfer. WL performed transmission electron microscopy. PR wrote the manuscript with assistance of KW and MC. All authors contributed to its finalization.

### Conflict of Interest Statement

The authors declare that the research was conducted in the absence of any commercial or financial relationships that could be construed as a potential conflict of interest.
