# Supplementary material for: Influence of the Core/Shell Structure of Indium Phosphide Based Quantum Dots on Their Photostability and Cytotoxicity
Source: Front Chem. 2019 Jun 27;7:466. doi: 10.3389/fchem.2019.00466 (PMC6610543; doi:10.3389/fchem.2019.00466)
Supplement: Supplementary file 1 [file Presentation_1.pdf]

Supporting Information for

Influence of the core/shell structure of indium phosphide based quantum dots on their photostability and cytotoxicity

K. David Wegner,[1,]* Fanny Dussert,[2] Delphine Truffier-Boutry,[3] Anass Benayad,[3] David Beal,[2] Lucia Mattera,[1] Wai Li Ling,[4] Marie Carrière,[2,]* Peter Reiss[1,]*

[1]Univ. Grenoble Alpes, CEA, CNRS, IRIG, SyMMES, STEP, 38000 Grenoble, France

[2]Univ. Grenoble Alpes, CEA, CNRS, IRIG, SyMMES, CIBEST, 38000 Grenoble, France

[3]Univ. Grenoble Alpes, CEA-LITEN L2N, 17 rue des Martyrs, 38000 Grenoble, France

[4]Univ. Grenoble Alpes, CEA, CNRS, IRIG, IBS, 38000 Grenoble, France

**Emails: wegner.karldavid@gmail.com, marie.carriere@cea.fr, peter.reiss@cea.fr*

Photophysical characterization of the QDs

Table S1: Summary of the photophysical properties of the InP-based QDs with different shells after the synthesis.

(in nm)	ZnSeS	ZnSeS/ZnS thin	ZnSeS/ZnS thick
1st Exciton peak	522	518	522
PL maxima	564	556	555
FWHM	63.2	57.5	57.1

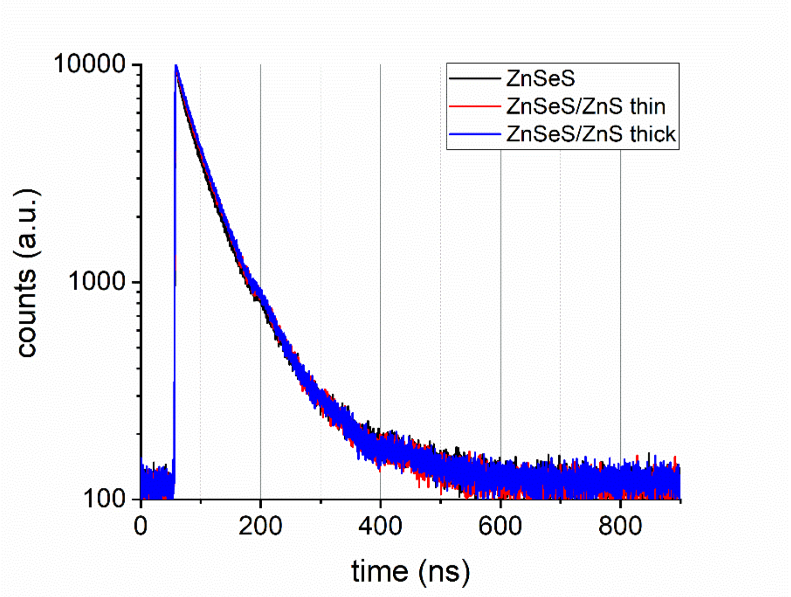

Figure S1: PL decay curves of InZnP QDs with ZnSeS (black), ZnSeS/ZnS thin (red), and ZnSeS/ZnS thick (black) shell.

Table S2: Decay times and intensity averaged decay time of InZnP QDs with different shells.

Shell	A_1	τ_1 (ns)	A_2	τ_2 (ns)	A_3	τ_3 (ns)	τ<int> (ns)
ZnSeS	2.4	4.9	43.9	36.0	53.7	78.1	**66.5**
ZnSeS/ZnS thin	1.4	5.7	53.3	39.7	45.3	82.2	**66.7**
ZnSeS/ZnS thick	0.9	4.2	53.3	39.5	45.9	79.0	**64.5**

Alumina-coated CSS QDs

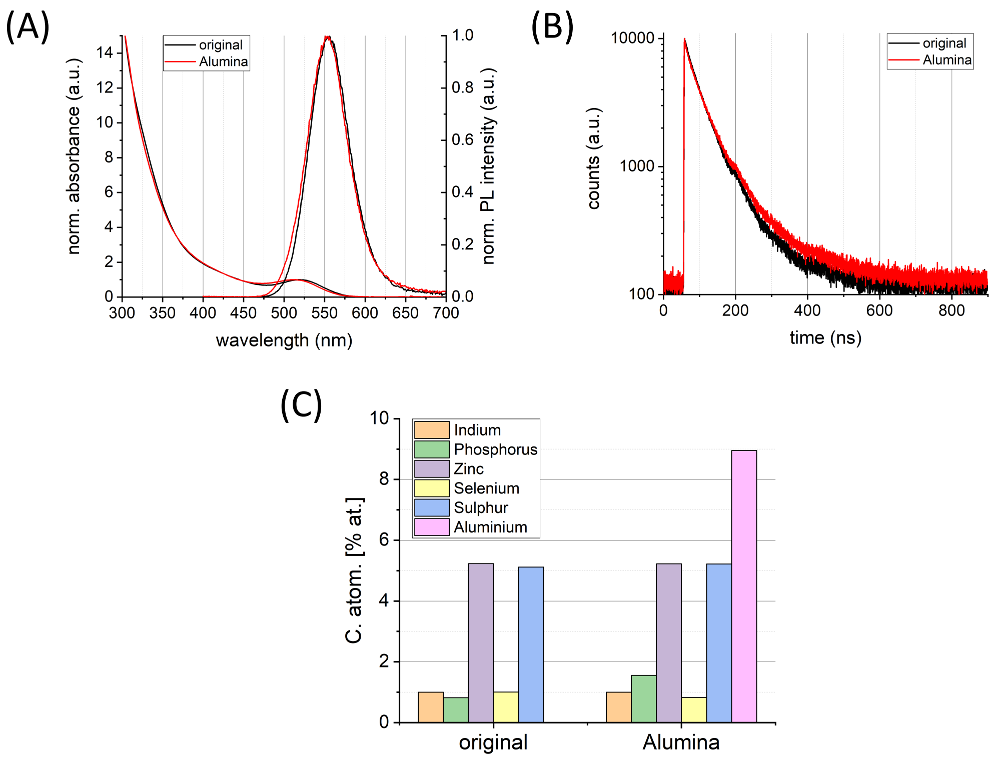

Figure S2: Comparison of normalized absorbance and PL spectra (A), PL decay curves (B), and EDX compositional analysis (C) of the InZnP/ZnSeS/ZnS thin QDs (original) and when using aluminum in the synthesis (Alumina).

Table S3: Decay times of the InZnP/ZnSeS/ZnS thin (original) and when using aluminum in the synthesis (Alumina).

Shell	A_1	τ_1 (ns)	A_2	τ_2 (ns)	A_3	τ_3 (ns)	τ<int> (ns)
original	1.4	5.7	53.3	39.7	45.3	82.2	**66.7**
Alumina	2.6	6.3	61.7	45.9	35.8	109.7	**82.8**

Photostability measurements in PMMA

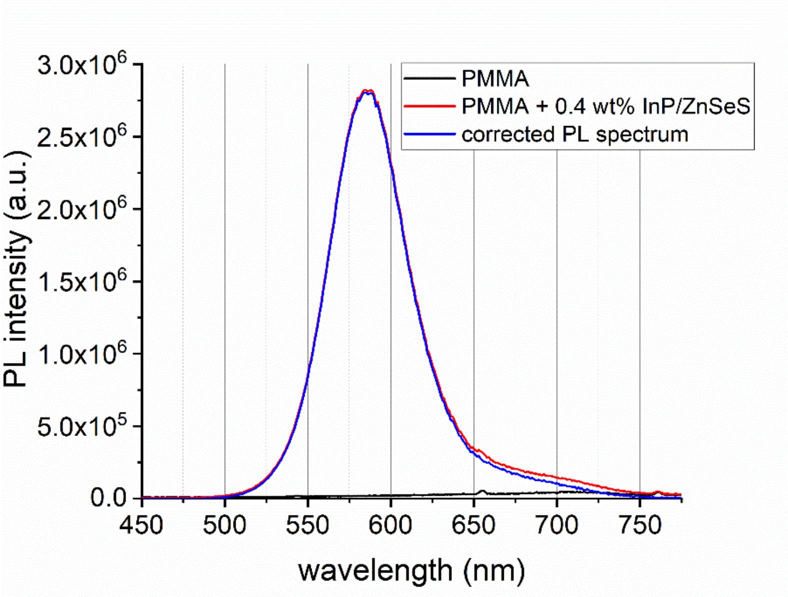

Figure S3: PL spectra of the pristine PMMA film (black), PMMA with 0.4 wt% InZnP/ZnSeS gradient shell QDs (red), and after subtraction of the PMMA reference signal (blue), demonstrating that the polymer matrix does not influence the PL properties.

Table S4: Quantum yield of the PMMA films with increasing InZnP/ZnSeS QD concentration.

[QD] (wt%)	Quantum yield (%)
0.05	3.2
0.075	3.4
0.1	4.2
0.2	4.1
0.4	5.3

(A)

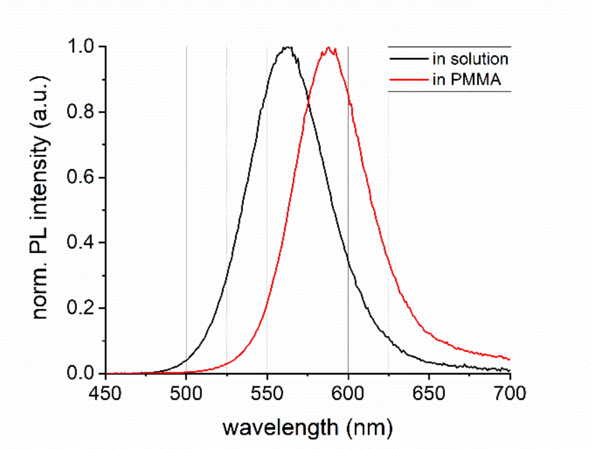

	In solution	In PMMA
PL maxima	**562 nm**	**588 nm**
FWHM	**56.8 nm**	**53.1 nm**
QY (%)	**50.9**	**7.6**

(B)

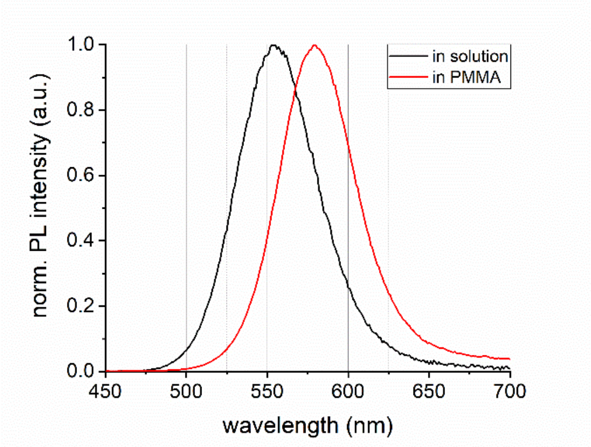

	In Hexane	In PMMA
PL maxima	**551 nm**	**565 nm**
FWHM	**58.9 nm**	**53.5 nm**
QY (%)	**48.8**	**10.1**

(C)

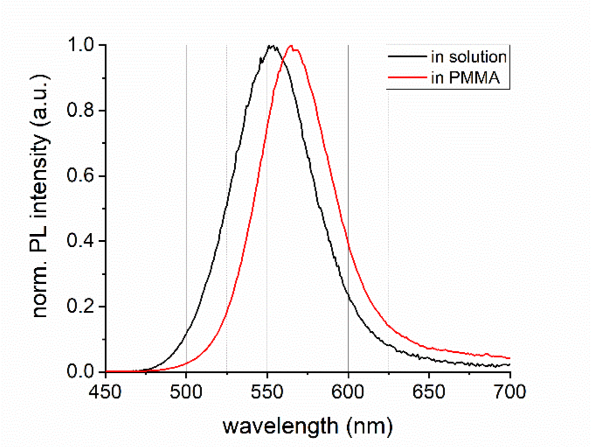

	In Hexane	In PMMA
PL maxima	**555 nm**	**579 nm**
FWHM	**57.2 nm**	**55.2 nm**
QY (%)	**64**	**16.9**

Figure S4: Comparison of the PL spectra and photophysical properties of InZnP/ZnSeS QDs (**A**), InZnP/ZnSeS/ZnS-alumina QDs (**B**), and InZnP/ZnSeS/ZnS (thick) QDs (**C**) in solution and embedded in the PMMA matrix.

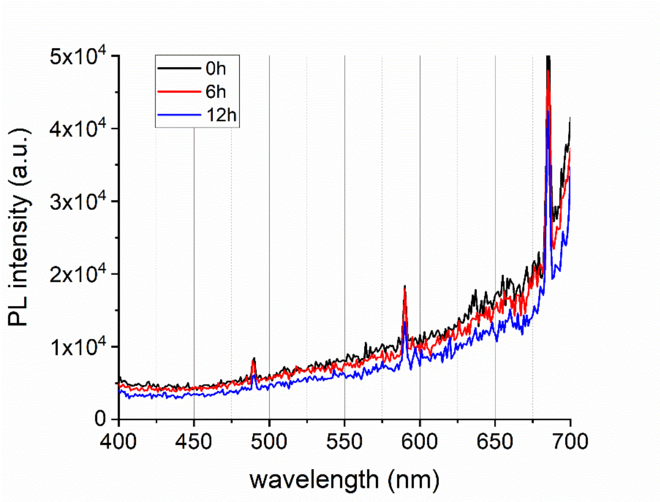

Figure S5: PL spectra of PMMA without QDs after 0h, 6h and 12h of illumination in the climate chamber.

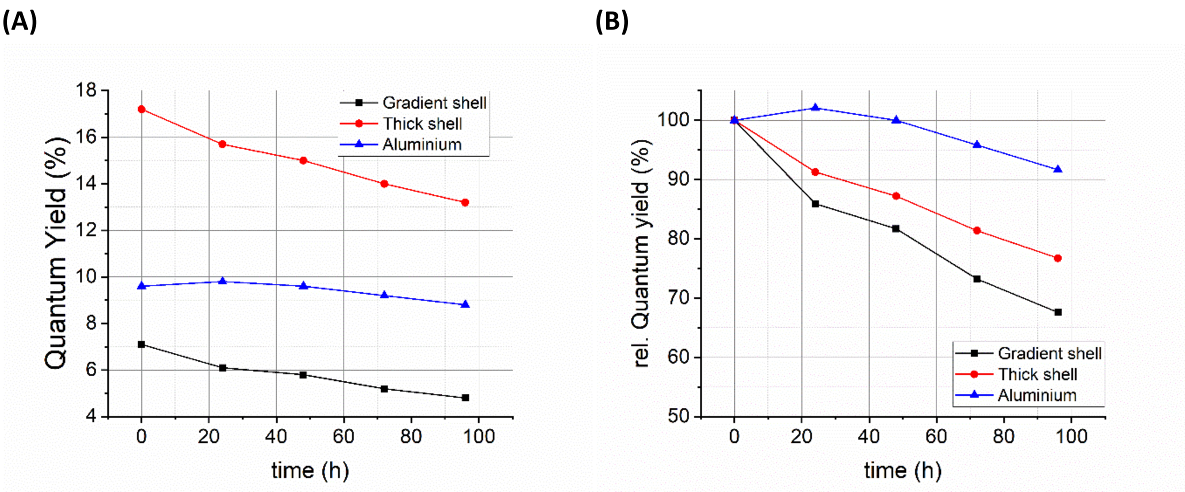

Figure S6: Evolution of quantum yield (A) and relative quantum yield (B) of the InZnP QDs with different shells embedded in PMMA kept in the dark and at room temperature.

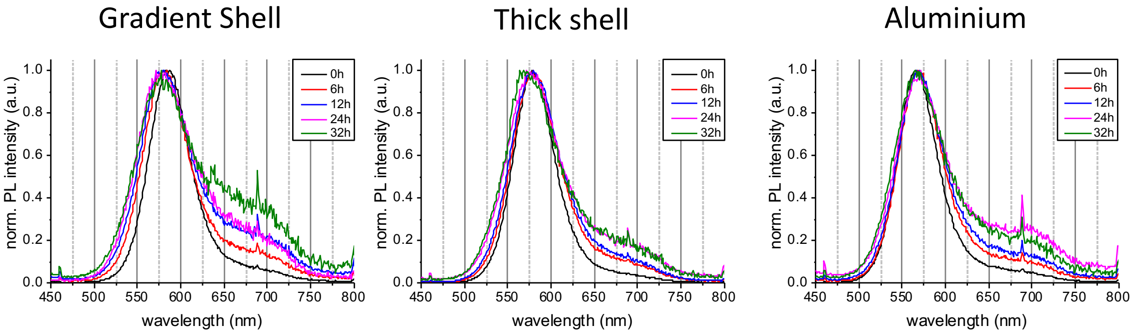

Figure S7: Evolution of the normalized PL spectra of the three systems during continuous irradiation in the aging chamber.

Table S5: Atomic concentrations determined by XPS.

	Atomic concentration			
	C-C	C-H	C-O	COO
Gradient 0h	96.3	0.5	0.7	2.5
Gradient 6h	83.5	8.0	3.4	5.1
Aluminium 0h	86.0	5.9	3.1	5.0
Aluminium 6h	82.2	7.0	4.1	6.7

	Atomic concentration						
	ZnS*	Zn	ZnSe*	ZnSSe*	In*S	$In_{(x)}O_{(y)}$	SO_4
Gradient 0h	24.7	29.9	13.0	---	13.4	4.9	14.0
Gradient 6h	18.7	34.4	11.8	---	5.3	13.6	16.2
Aluminium 0h	21.7	59.3	5.2	4.7	4.0	5.1	---
Aluminium 6h	22.0	56.1	5.0	5.8	3.2	7.9	---

Phase transfer with Penicillamine

Table S6: Summary of the photophysical properties of the InZnP QDs with different shell before (original) and after phase transfer (Pen).

	**ZnSeS**		**ZnSeS/ZnS thin**		**ZnSeS/thick**	
**(in nm)**	**original**	**Pen**	**original**	**Pen**	**original**	**Pen**
1st Exciton peak	**522**	**518**	**518**	**518**	**522**	**518**
PL maxima	**564**	**578**	**556**	**568**	**555**	**564**
FWHM	**63.2**	**66.2**	**57.5**	**61.3**	**57.1**	**60**
Stokes shift	**42**	**60**	**38**	**50**	**33**	**46**

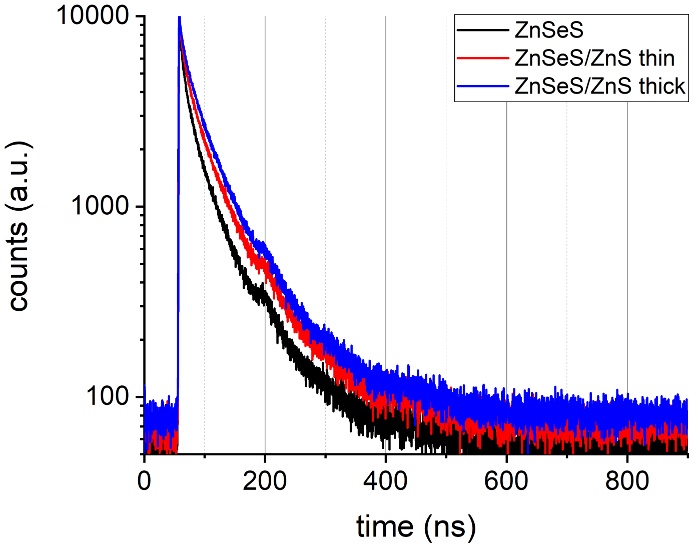

Figure S8: PL decay curves of InZnP QDs with different shells after phase transfer using penicillamine.

Table S7: Decay times and intensity averaged decay time of InZnP QDs with different shells after phase transfer.

Sample	A_1	τ_1 (ns)	A_2	τ_2 (ns)	A_3	τ_3 (ns)	τ<int> (ns)
ZnSeS	8.8	3.3	41.2	22.1	50.0	68.8	**58.7**
ZnSeS/ZnS thin	6.4	4.0	42.3	27.7	51.4	75.3	**63.9**
ZnSeS/ZnS thick	4.8	4.2	40.8	29.6	54.4	73.6	**63.2**

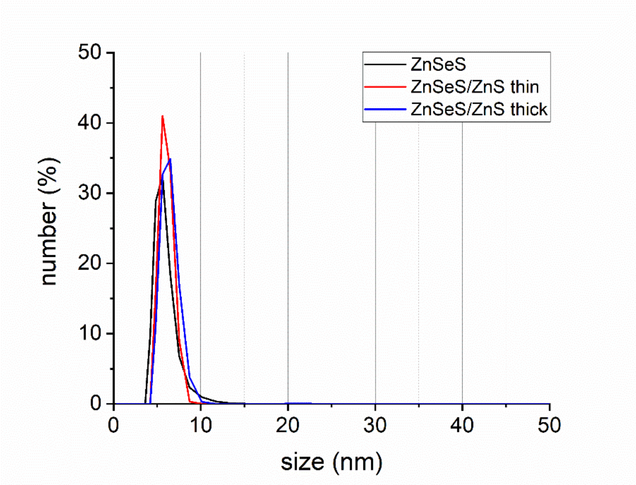

Figure S9: DLS measurements of the phase transferred InZnP QDs with different shells.

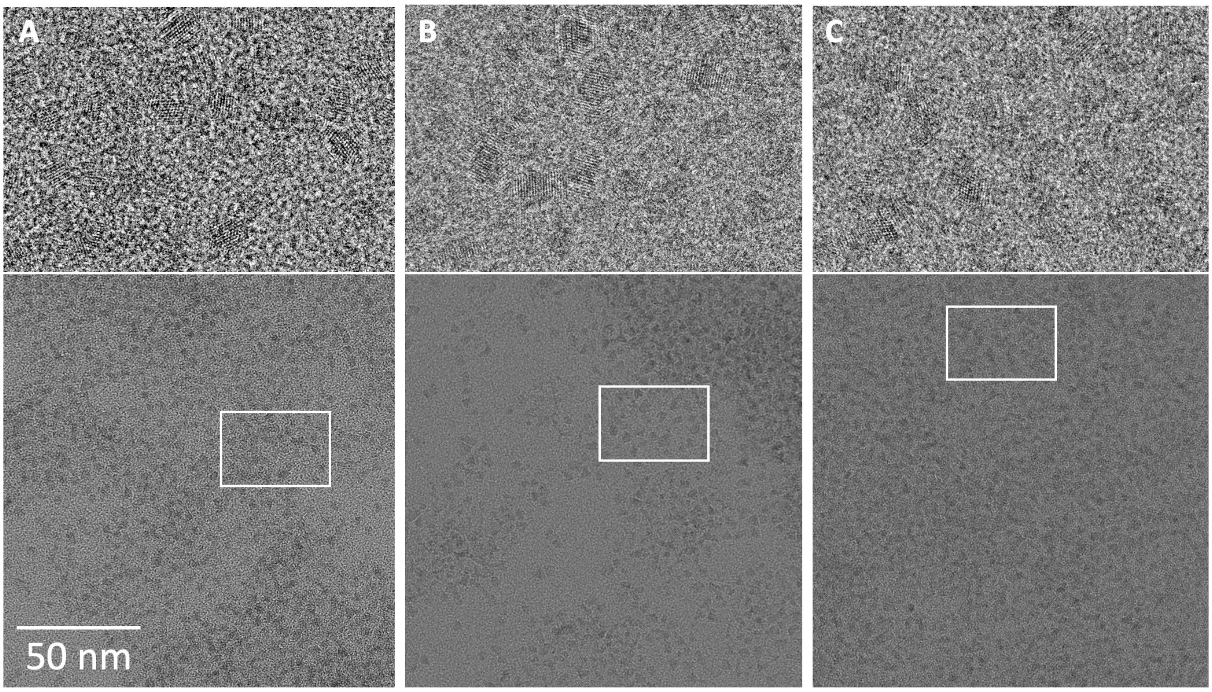

Figure S10: TEM images at different magnification of the different core/shell systems: A) InZnP/ZnSeS gradient shell, B) InZnP/ZnSeS/ZnS thin, C) InZnP/ZnSeS/ZnS thick.
